# Stressful Presentations: Mild Cold Stress in Laboratory Mice Influences Phenotype of Dendritic Cells in Naïve and Tumor-Bearing Mice

**DOI:** 10.3389/fimmu.2014.00023

**Published:** 2014-02-10

**Authors:** Kathleen M. Kokolus, Haley M. Spangler, Benjamin J. Povinelli, Matthew R. Farren, Kelvin P. Lee, Elizabeth A. Repasky

**Affiliations:** ^1^Department of Immunology, Roswell Park Cancer Institute, Buffalo, NY, USA

**Keywords:** cold stress, thermoregulation, norepinephrine, mouse models of cancer, anti-tumor immunity

## Abstract

The ability of dendritic cells (DCs) to stimulate and regulate T cells is critical to effective anti-tumor immunity. Therefore, it is important to fully recognize any inherent factors which may influence DC function under experimental conditions, especially in laboratory mice since they are used so heavily to model immune responses. The goals of this report are to 1) briefly summarize previous work revealing how DCs respond to various forms of physiological stress and 2) to present new data highlighting the potential for chronic mild cold stress inherent to mice housed at the required standard ambient temperatures to influence baseline DCs properties in naïve and tumor-bearing mice. As recent data from our group shows that CD8^+^ T cell function is significantly altered by chronic mild cold stress and since DC function is crucial for CD8^+^ T cell activation, we wondered whether housing temperature may also be influencing DC function. Here we report that there are several significant phenotypical and functional differences among DC subsets in naïve and tumor-bearing mice housed at either standard housing temperature or at a thermoneutral ambient temperature, which significantly reduces the extent of cold stress. The new data presented here strongly suggests that, by itself, the housing temperature of mice can affect fundamental properties and functions of DCs. Therefore differences in basal levels of stress due to housing should be taken into consideration when interpreting experiments designed to evaluate the impact of additional variables, including other stressors on DC function.

## Introduction

Dendritic cells (DCs) play a vital role in the generation of effective and long-term immune protection from cancer and other diseases. DCs are antigen presenting cells, which educate tumor-specific T cells and provide signals for T cell proliferation and expansion (1, 2). Importantly, DCs bridge the innate and adaptive immune responses so their presence and functional capacity affect both arms of anti-tumor immunity (3, 4). Properties of DCs that are investigated to determine their stage of development include surface expression of major histocompatibility complex (MHC) class II molecules and co-stimulatory CD86 as well as cytokine production. Additionally, DCs are also being used clinically in cancer vaccines (5, 6) and this approach has rendered promising results; however, considerable room for improvement remains (7–9).

In addition to anti-tumor immunity and immune surveillance, DCs also participate in tolerizing the immune system to tumor antigens, which can render the anti-tumor immune response ineffective (10). Cross-presentation, a process that DCs undergo in order to activate CD8^+^ T cells, plays a major role in generating anti-tumor immunity (11), however; when DCs of tumor-bearing hosts undergo this vital process, T cell tolerance often results (5). Recently, it has been reported that DCs able to up-regulate MHC II (signal 1) in the absence of CD86 (signal 2) become tolerogenic DCs (12, 13). Although considerable progress has been made toward understanding how DCs become tolerogenic (10, 14, 15), the precise mechanisms by which tumors modulate cross-priming to suppress the CD8^+^ T cell response remain largely unknown. This incomplete understanding of the role DCs play in immune evasion remains a vital question as DCs are being actively investigated in mouse models to help reveal their role in the anti-tumor immune response. Therefore, it is important to fully recognize the impact of any inherent physiological factors in mice, which can alter DC function and to understand the impact these factors could have on experimental models of antigen presentation and immunotherapy.

We have been interested in the effects of various types of biologically relevant stress on the functional properties of immune cells (16) and have previously reported on the impact of mild (fever-range) heat stress on DC function (17, 18). It is important to note that there are a wide variety of stressors including physical, environmental, and emotional forms of stress that can alter homeostasis in cells or in the whole organism (19). Two major hormonally driven mechanisms are believed to mediate the influence of stress on the immune response. Glucocorticoids are released following stress leading to increased glucose metabolism necessary to provide extra energy to combat that stressor. Additionally, catecholamines, such as norepinephrine (NE), are released from sympathetic nerves and bind receptors on immune cells thereby impacting the immune response. Both of these hormonal mediators can influence immune processes including cell proliferation, migration, and cytokine production (20). Here, we first briefly summarize some of the previous work done to investigate the more specific effects of stress on DC function. While some studies show that acute, short term, stress may enhance DC function *in vitro*, resulting in a better ability to prime naïve T cells, other studies, particularly those which utilize the addition of exogenous stress hormones, reveal that stress impedes DC function *in vivo*. We outline reports suggesting a vital role of the stress hormone NE on DC function *in vivo* but not *in vitro*. We also summarize literature showing beneficial effects of a mild thermal stress on DC function both *in vitro* and *in vivo*. Finally, we report that when mice used to investigate DC function are housed at standard ambient temperatures they experience an underappreciated form of chronic physiological cold stress that alters the baseline used to understand the impact of experimental stressors or other treatments on DC function. We suggest that chronic mild cold stress, similar to other forms of stress inherent to mouse caging conditions including stress caused by lack of exercise and overeating (21), should be taken into consideration when assessing baseline properties of DCs in naïve or tumor-bearing mice.

## Stress Can Target DC Function

Dendritic cells have already been the subject of many studies investigating the impact of stress on immune function. Acute stressors, lasting minutes to hours, have been shown to augment DC function as seen by enhanced maturation and increased trafficking from skin to lymph nodes (22, 23). Prior to immunization, specific kinds of acute stress, such as psychological stress induced by placing mice in restraints or on a slow moving shaker works as an adjuvant leading to increased DC migration from the skin to the lymph nodes and also improves antigen-specific T cell priming (24, 25). The impact of such acute psychological stress on DCs has also been investigated in humans. Social stress in human participants (induced by public speaking) generates a decrease in skin DCs, which the authors suggest indicates that these cells have trafficked to the lymph node (26) where they are available to interact with T cells and initiate immune activation. However, while some stressors may elicit beneficial effects on DC function and general immunity, chronic or excessive exposure to stress is generally thought to negatively influence immune function (27). Many studies, particularly those using exogenous administration of glucocorticoids, stress hormones which signal to turn down immune activity, suggest inhibitory effects of stress on DC function (28, 29). Both oral (30) and topical (31) application of glucocorticoids leads to a marked reduction in DC numbers. Many studies specifically investigate the impact of dexamethasone (DEX), a commonly prescribed glucocorticoid, on DC development and function. It has been shown that DEX greatly reduces epidermal DC numbers in mice (32, 33) as well as in the spleen, lymph node, and liver (34, 35). DEX treatment also limits DC migration to the draining lymph node (36). Additionally, DEX is correlated with reduced expression of surface maturation markers on DCs including CD86 and MHC class II (35, 37, 38). *In vitro*, DEX treatment reduces the ability of bone marrow (39, 40) and skin derived DCs (32, 41) as well as a murine epidermal DC line (42) to stimulate T cells. DEX also impairs antigen presentation by DCs reducing T cell activation *in vivo* (35). It has also been shown that following DEX treatment, DCs are unable to fully mature and these immature DCs induced a subpopulation of immunosuppressive regulatory T (T_reg_) cells (39). Additionally a reduction of interleukin (IL)-1β and IL-12p70 secretion from DCs has been shown following DEX treatment (37). Compared to control cells, glucocorticoid-treated DCs produce less granulocyte macrophage colony-stimulating factor (GM-CSF), tumor necrosis factor-alpha (TNF-α) and IL-1α, all cytokines required for survival and maturation, and are less apt to initiate antigen presentation and migration (43). Further, treatment with other glucocorticoids (hydrocortisone or clobetasol) led to DC apoptosis identified by DNA damage, caspase-3 activity, and CD95 up-regulation (43).

Catecholamines, such as NE and epinephrine, also play an important role in mediating the relationship between stress and DCs. Manipulating the function of stress induced catecholamines has been linked to altered DC function (44). For example, when healthy patients were administered a β-adrenergic agonist (oral salbutamol), which mimics NE signaling, IL-12 production by DCs was decreased, inhibiting Th1 development (45). Another study found that NE similarly suppressed IL-12 production in a dose-dependent manner and that this was reversible with a glucocorticoid agonist, RU 486 (46). As Th1 and Th2 responses are mutually inhibitory, this leads to an increasingly prominent Th2 environment, which is defined by various immunosuppressive properties including inhibition of macrophage activation, T cell proliferation, and pro-inflammatory cytokine production (47). The effects of catecholamines may be most important to DCs in the early stages of antigen processing (44). Short term exposure of bone marrow-derived DCs to NE or epinephrine at the early stage of stimulation inhibits IL-12 and favors IL-10 production as well as a reduced ability to stimulate T cells (48). Skin DCs are also sensitive to catecholamine signaling. *In vitro*, treatment with NE, epinephrine, or β-adrenergic agonist (isoproterenol) hindered skin DCs from presenting antigen and this effect was reversed by treatment with ICI 118,551, a β_2_-adrenergic antagonist (49). DC migration is NE dependent as demonstrated by decreased DC migration *in vivo* following NE depletion with 6-hydroxydopamine treatment (25). Additionally, NE has been shown to enhance phosphatidylinositol 3-kinase mediated antigen uptake by DCs (50). Taken together, these reports suggest that although some types of stress may benefit DCs under certain circumstances, it is generally accepted that chronic stress dampens many aspects of DC function.

## Effects of Mild Hyperthermia on DCs

Environmental conditions have long been manipulated to create physiologically relevant stress. Thermal stress, induced when environmental conditions are either too hot or too cold to allow basal metabolism to maintain normal body temperature, is a classically studied stress in mice and humans (51). While conditions of severe heat or cold can be quite damaging to immunity, mild heat stress has been studied for its positive effects since ancient times because of its potential relationship to fever (52–55). In response to infection, body temperature increases to varying extents among different animals, but in all cases, homeostatic functions shift toward producing and conserving heat (52). Generally, temperature elevation during fever ranges between 1 and 5°C above normal body temperatures (56–58). The physiological effects of fever have been mimicked experimentally by using mild hyperthermia treatments in mice, where body temperature is temporarily raised to fever-range (16). Many studies, including those from our own group, have examined how mild hyperthermia affects DCs and their function (17, 59–64).

Dendritic cell maturation is determined by the up-regulation of surface markers including MHC class II and CD86 (65, 66). Mild thermal stress increases levels of both of these markers on DCs (59). *In vitro* heating accelerates DC maturation as demonstrated by up-regulation of both CD86 and MHC class II (60, 61, 67). *In vivo* studies have also shown that up-regulation of both MHC class II and CD86 molecules on the surface of DCs from mice treated with whole body hyperthermia (61, 68). Additionally, hyperthermia in combination with other treatments including ionizing radiation (69), magnetic nanoparticles (70, 71), radiofrequency ablation (72) and vaccination (73, 74) results in enhanced DC function.

Dendritic cell migration to the lymph node is an important function required for efficient antigen presentation and our group and others have shown that mild heat stress can promote migratory activity of DCs. DCs in ear skin subjected to thermal stress in culture show increased migration compared to control samples (75), while increased DC migration into the lymph nodes of thermally stressed mice has also been demonstrated (61).

Heat treatment results in improved stimulatory function of DCs (59, 63, 67). Heated OVA-loaded DCs induce greater interferon-gamma (IFN-γ) responses from SINFEKL-specific T cells (64). Heat-treated SINFEKL pulsed DCs elicit greater antigen-specific CD8^+^ T cell proliferation than unheated DCs (67). Heat also enhances the ability of DCs to cross-present to CD8^+^ T (76) and activates CD4^+^ T cells leading to antigen dependent memory (77). Additionally, mild hyperthermia alters the production of cytokines and chemokines from DCs, which are important for ensuring effective T cell priming. Mild heating increases DC production of inflammatory cytokines including IFN-γ, IL-17, IL-10, IL-12, and TNF-α (60, 61, 63). Taken together, the growing body of literature describing the effects of mild heat stress on DCs indicates that mild heat stress enhances DC function by promoting maturation and migration and increasing inflammatory cytokine production to assist with mediation of T cell priming to elicit T cell proliferation.

## Effects of Cold Stress on DCs

We have summarized some of the previously reported complex effects of stress on DCs, including the general beneficial effects of temporary mild hyperthermia. We wondered whether the baseline function of DCs in these types of studies is influenced by ambient temperature used to house mice in research facilities. Laboratory mice are under a mild, yet constant cold stress as they are group housed at a cool (sub-thermoneutral) temperature (78–80). Additionally, since laboratory mice are provided with unlimited access to food and housed in small cages, which do not allow adequate room to exercise, they also experience additional metabolic stresses (21). Although these stressors have been identified as being important in other fields of research, such as obesity (81), they are not generally accounted for in the field of cancer immunology.

The fact that mice are mildly, yet chronically, cold stressed is not determined simply by body temperature measurements. In fact, while body temperature appears normal (~37°C) for mice housed at standard ambient temperatures required for research facilities (55), thermal preference studies over many decades have shown that mice prefer a warmer housing temperature near thermoneutrality (57, 78, 82, 83) indicating the degree of cold stress prompted by such housing. The degree to which underlying chronic cold stress has impacted the interpretation of the effects of other types of stress on immune function remains to be determined. Importantly, NE is released in response to stressors, including cold stress and, as detailed above, has a very significant influence on DC function.

Recent literature has detailed the impact of chronic cold stress in mice. The relationship between cold stress and metabolism has been investigated and alterations in insulin production (84), NE secretion (85), function of uncoupling proteins (81), and energy expenditure (86, 87) have been identified. Developmental and behavioral effects including differences in limb and tail length (88), cardiac tone and heart rate (89), and sleep (90) have also been observed when comparing cold stressed to non-stressed mice. Most recently, our group has shown that mild cold stress associated with standard housing conditions negatively impacts CD8^+^ T cell dependent anti-tumor immune responses (55). To test whether DC function is influenced by chronic cold stress, we studied the impact of sub-thermoneutral housing temperatures on DC phenotype and function comparing the results to that seen from mice housed at thermoneutrality. Importantly, core body temperature in both groups of mice is the same, as shown previously (55).

**Figure 1:**
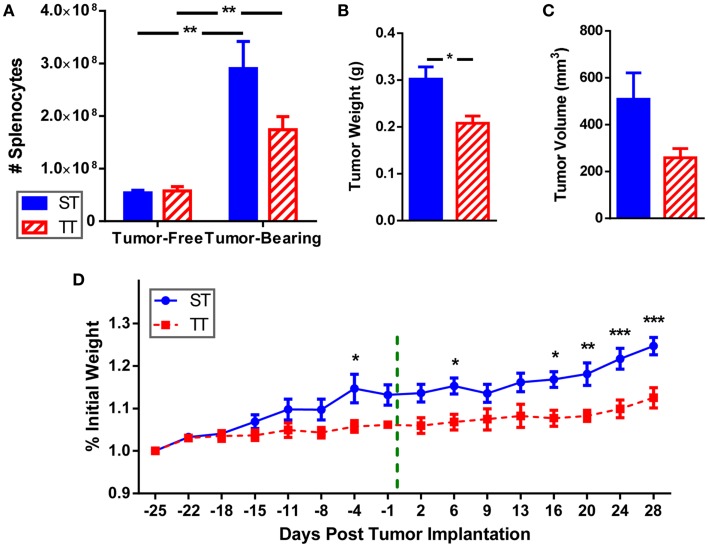
**Splenocytes, tumor size, and body weight are increased when mice are maintained at ST compared to TT**. 4T1 tumor-bearing BALB/c mice and age-matched controls were maintained at ST or TT. **(A)** Splenocytes obtained from control and tumor-bearing mice were counted and **(B)** tumor weight and **(C)** volume were measured. Data presented as mean ± SEM; *n* = 5/group; Student’s *t*-test; *p < 0.05, **p < 0.01. **(D)** Change in weight from the start of the experiment was measured. - - - - indicates day of tumor inoculation. Data presented as mean ± SEM; *n* = 5/group; two-way ANOVA with Bonferroni post-tests; *p < 0.05, **p < 0.01, ***p < 0.001.

We examined splenocytes from tumor-free and 4T1 tumor-bearing mice housed at standard (ST; 22°C) and thermoneutral (TT; 30°C) temperature. Because at 30°C the metabolic cold stress is greatly reduced, these mice represent un-cold stressed animals whereas their counterparts at 22°C are under chronic cold stress. We found that the number of splenocytes is similar in naïve (tumor-free) mice at ST and TT (Figure 1A). However, inoculation of mice with tumors induces an increase in splenocyte number at both ambient temperatures, however, this increase is larger at ST than at TT (Figure 1A). Confirming previous data (55), tumors grew slower in mice at TT compared to ST; tumor weight (Figure 1B) and volume (Figure 1C) were reduced in TT mice compared to ST mice. We also examined body weight for mice housed at each ambient temperature and found that prior to tumor inoculation mice at ST gained weight faster than mice at TT (Figure 1D). As tumors began to grow, mice at ST continued to gain even more weight than mice at TT (Figure 1D). These data show that animals housed at TT are physically smaller than those mice used as standard control models, while 4T1 tumor growth is accelerated in ST control mice.

**Figure 2:**
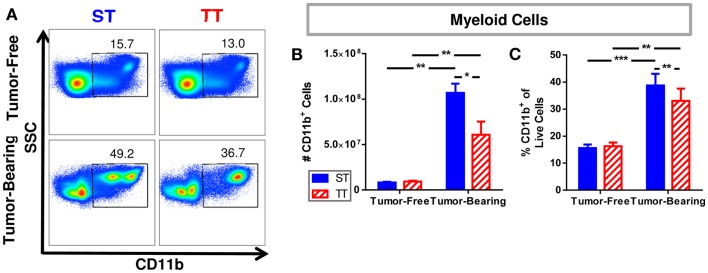
**Splenic myeloid cells are increased in tumor-bearing mice maintained at ST compared to TT**. Single cell suspensions of splenocytes from 4T1 tumor-bearing mice and age-matched controls were stained for CD11b and analyzed by flow cytometry. **(A)** Representative dot plots from each group show the gating strategy used to select CD11b^+^ cells. Percentage of cells are shown above their respective gate. **(B)** The absolute number of CD11b^+^ cells calculated from the total number of splenocytes counted in each individual mouse. **(C)** The percentage of CD11b^+^ cells of the total population of live cells as determined by DAPI staining. Data presented as mean ± SEM; *n* = 5/group; Student’s *t*-test; *p < 0.05, **p < 0.01, ***p < 0.001.

We previously reported that spleens from mice at TT have fewer CD11b^+^GR-1^+^ myeloid derived suppressor cells (MDSCs) (55), so we wondered whether pan myeloid cells (CD11b^+^) were similarly impacted by temperature (Figure 2A). We first determined that tumor-bearing mice at ST have significantly more CD11b^+^ myeloid cells as well as a higher percentage of CD11b^+^ cells compared to tumor-bearing mice at TT (Figure 2B). The number and proportion of splenic myeloid cells in tumor-free animals was unchanged (Figure 2C). These results suggest that the effects of 4T1 tumor growth on the accumulation of myeloid cells in the spleen may be overestimated in mice housed under standard conditions since the cellular increase is also dependent on ambient temperature.

**Figure 3:**
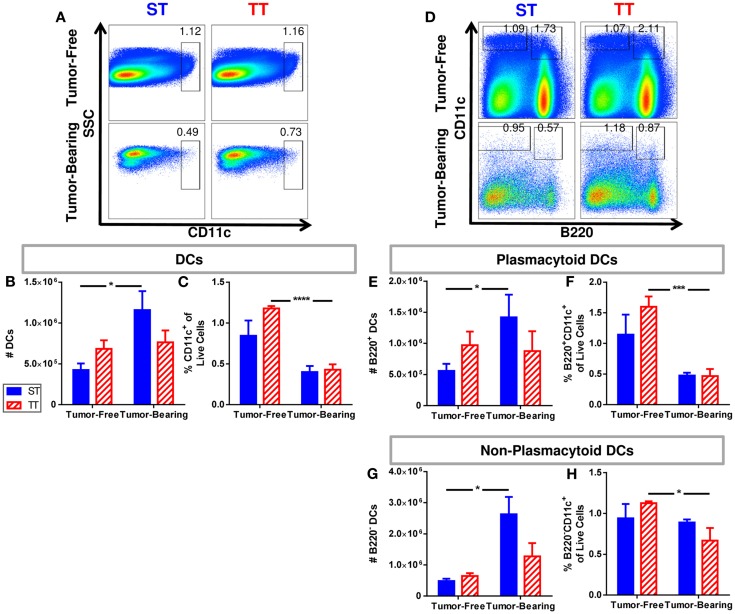
**Tumor-bearing mice maintained at ST have an increased frequency of DCs compared to those at TT**. Single cell suspensions of splenocytes from 4T1 tumor-bearing mice and age-matched controls were stained for CD11c and **(D–H)** B220 and analyzed by flow cytometry. **(A)** Representative dot plots from each group show the gating strategy used to select CD11c^+^ cells. Percentage of cells are shown above their respective gate. **(B)** The absolute number of CD11c^+^ cells calculated from the total number of splenocytes counted in each individual mouse. **(C)** The percentage of CD11c^+^ cells of the total population of live cells as determined by DAPI staining. **(D)** Representative dot plots from each group show the gating strategy used to select B220^+^CD11c^+^ and B220^−^CD11c^+^ cells. Percentage of cells are shown above their respective gate. **(E)** The absolute number of B220^+^CD11c^+^ cells calculated from the total number of splenocytes counted in each individual mouse. **(F)** The percentage of B220^+^CD11c^+^ cells of the total population of live cells as determined by DAPI staining. **(G)** The absolute number of B220^−^CD11c^+^ cells calculated from the total number of splenocytes counted in each individual mouse. **(H)** The percentage of B220^−^CD11c^+^ cells of the total population of live cells as determined by DAPI staining. Data presented as mean ± SEM; *n* = 5/group; Student’s *t*-test; *p < 0.05, ***p < 0.001, ****p < 0.0001.

It has been reported that DC numbers in cancer patients are reduced compared to healthy controls (91); thus, we next investigated numbers of splenic DCs in tumor-free and tumor-bearing mice maintained at ST and TT based on CD11c expression. Total DCs were identified as CD11c^+^ cells. We found that absolute numbers of splenic DCs (Figure 3A) increased following tumor implantation in mice at ST but not at TT (Figure 3B). However, the proportion of DCs decreased at both ST and TT following tumor inoculation (Figure 3C). We next examined plasmacytoid DCs (B220^+^CD11c^+^) (Figure 3D) which, following stimulation, are major interferon producers (92). We discovered that absolute numbers of plasmacytoid DCs increase following tumor inoculation in mice at ST but not at TT (Figure 3E), whereas percentages significantly decrease following tumor inoculation in mice at TT but not at ST (Figure 3F). When we investigated non-plasmacytoid DCs (B220^−^CD11c^+^) (Figure 3D) (93–95), we again found that absolute numbers increase following tumor inoculation in mice at ST but not at TT (Figure 3G) but that percentages of these cells significantly decrease following tumor inoculation in mice at TT only (Figure 3H). These data demonstrate that the number of DCs found in the spleens of laboratory mice do not show the expected increase in numbers after tumor inoculation when mice are maintained at thermoneutrality. Thus, ambient temperature should be considered when interpreting data regarding immune cell subsets in the spleens from mice used for cancer immunology studies.

**Figure 4:**
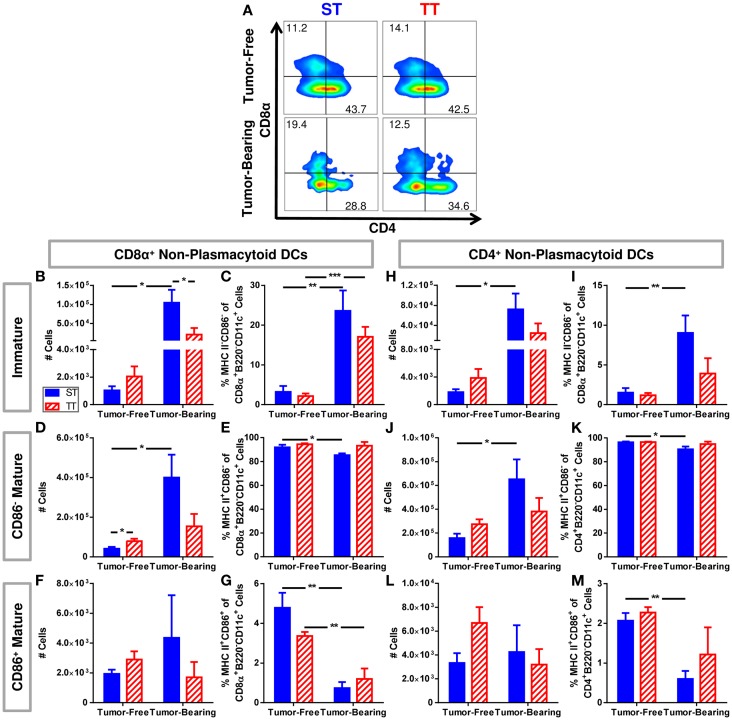
**Tumor-bearing mice maintained at ST have proportionally more non-plasmacytoid DCs than those at TT, but these DCs primarily display an immature phenotype**. Single cell suspensions of splenocytes from 4T1 tumor-bearing mice and age-matched controls were stained for CD8α, CD4, CD11c, MHCII, CD86 and analyzed by flow cytometry. **(B–G)** Quantification of data describing CD8α^+^ non-plasmacytoid DCs. **(A)** Representative dot plots from each group show the gating strategy used to select CD4 and CD8α cells from the non-plasmacytoid parent population shown in Figure 3D. Percentage of cells are shown above their respective gate. **(B)** The absolute number of CD8α^+^MHC II^−^CD86^−^ B220^−^CD11c^+^ cells calculated from the total number of non-plasmacytoid cells. **(C)** The percentage of MHC II^−^CD86^−^ cells of the total population of CD8α^+^ non-plasmacytoid cells. **(D)** The absolute number of CD8α^+^MHC II^+^CD86^−^B220^−^CD11c^+^ cells calculated from the total number of non-plasmacytoid cells. **(E)** The percentage of MHC II^+^CD86^−^ cells of the total population of CD8α^+^ non-plasmacytoid cells. **(F)** The absolute number of CD8α^+^MHC II^+^CD86^+^B220^−^CD11c^+^ cells calculated from the total number of non-plasmacytoid cells. **(G)** The percentage of MHC II^+^CD86^+^ cells of the total population of CD8α^+^ non-plasmacytoid cells. **(H–M)** Quantification of data describing CD4^+^ non-plasmacytoid DCs. **(H)** The absolute number of CD4^+^MHC II^−^CD86^−^B220^−^CD11c^+^ cells calculated from the total number of non-plasmacytoid cells. **(I)** The percentage of MHC II^−^CD86^−^ cells of the total population of CD4^+^ non-plasmacytoid cells. **(J)** The absolute number of CD4^+^MHC II^+^CD86^−^B220^−^CD11c^+^ cells calculated from the total number of non-plasmacytoid cells. **(K)** The percentage of MHC II^+^CD86^−^ cells of the total population of CD4^+^ non-plasmacytoid cells. **(L)** The absolute number of CD4^+^MHC II^+^CD86^+^B220^−^CD11c^+^ cells calculated from the total number of non-plasmacytoid cells. **(M)** The percentage of MHC II^+^CD86^+^ cells of the total population of CD4^+^ non-plasmacytoid cells. Data presented as mean ± SEM; *n* = 5/group; Student’s *t*-test; *p < 0.05, **p < 0.01, ***p < 0.001.

We further dissected the non-plasmacytoid cell population by quantifying a subset of immature (MHCII^−^CD86^−^) and two subsets of mature (CD11c^+^MHCII^+^CD86, CD11c^+^MHCII^+^CD86^−^) cells among CD8α^+^ and CD4^+^ non-plasmacytoid DCs (Figure 4A). CD8α^+^ DCs are major producers of IL-12, able to initiate a robust inflammatory response as well as efficiently presenting antigen to CD8^+^ T cells (96–99). We found that both absolute numbers (Figure 4B) and percentages (Figure 4C) of immature CD8α^+^ non-plasmacytoid DCs are increased to a greater extent following tumor inoculation in mice at ST compared to TT. Absolute numbers of CD86^−^ mature CD8α^+^ non-plasmacytoid DCs increased following tumor inoculation in mice at ST but not TT (Figure 4D). The percentage of CD86^−^ mature CD8α^+^ non-plasmacytoid DCs decreased at ST but not TT following tumor inoculation (Figure 4E). CD86^+^ mature CD8α^+^ non-plasmacytoid DCs were unchanged in absolute number (Figure 4F) but their proportion in the spleen was modestly, yet significantly decreased at both ST and TT following tumor inoculation (Figure 4G). The increased numbers of immature and CD86^−^ mature CD8α non-plasmacytoid DCs present in mice at ST suggests that many of the DCs from these mice may not be able to become activated.

Further, we investigated the same subsets of immature and mature CD4^+^ non-plasmacytoid DCs (Figure 4A). We also found a major increase in absolute numbers (Figure 4H) and percentage (Figure 4I) of immature CD4^+^ non-plasmacytoid DCs in mice at ST but not TT following tumor inoculation. At ST, there was an increase in absolute number (Figure 4J) and a decrease in the percentage (Figure 4K) of CD86^−^ mature CD4^+^ non-plasmacytoid DCs in response to tumor, but no significant changes were observed at TT. Again we saw no changes in absolute numbers of CD86^+^ mature CD4^+^ non-plasmacytoid DCs at either temperature (Figure 4L). We did see a reduced percentage of CD86^+^ mature CD4^+^ non-plasmacytoid DCs at ST but not at TT following tumor inoculation (Figure 4M). Interestingly, despite the increased number of non-plasmacytoid DCs in mice at ST (Figure 3G), there are no differences in the number of mature DCs (Figures 4F,L) suggesting that although DC numbers appear to be increased in cold stressed mice, many of these cells are unable to become activated in the presence of a 4T1 tumor.

**Figure 5:**
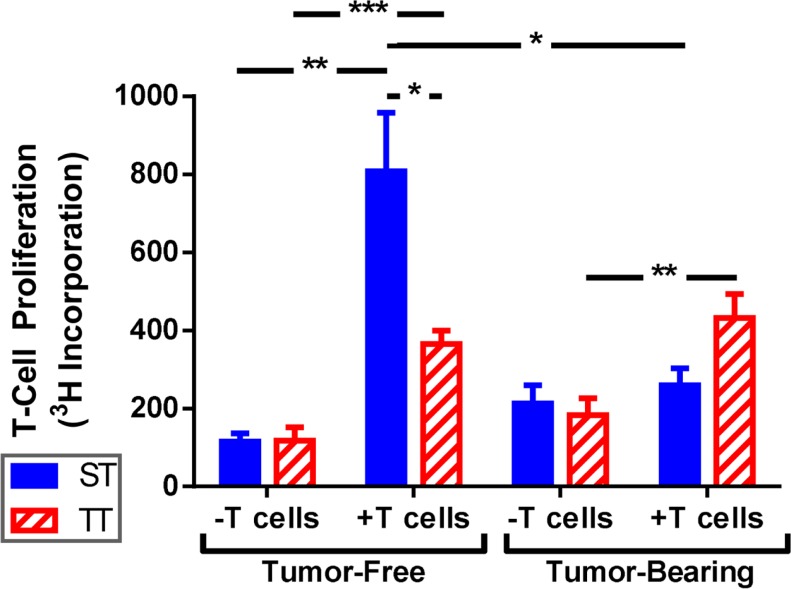
**T cells are activated better by splenocytes from mice at TT than ST**. Total splenocytes from tumor-free and tumor-bearing BALB/c mice and lymphocytes from C57BL/6 mice were cultured 1:2. T cell proliferation was measured by ^3^H-thymidine incorporation. Data presented as mean ± SEM; *n* = 5/group; Student’s *t*-test; *p < 0.05, **p < 0.01,***p < 0.001.

Due to the increased overall numbers but relatively low number of mature splenic DCs seen in mice at ST, we asked whether DCs from mice at ST were impaired at antigen presentation and their ability to activate naïve T cells. To answer this question, we performed mixed lymphocyte reactions using irradiated splenocytes from ST and TT tumor-free and tumor-bearing mice as stimulator cells and T cells from naïve ST mice as the responders. Responder and stimulator cells were co-cultured at a 2:1 ratio for 72 h and then T cell proliferation was measured by ^3^H-thymidine incorporation. As expected, we found that stimulator cells from tumor-free mice at both ST and TT were able to induce significant T cell proliferation (Figure 5; tumor-free). However, stimulator cells from tumor-free mice at ST elicited significantly more T cell proliferation than those from mice at TT (Figure 5; tumor-free, + T cells). Interestingly, when we looked at tumor-bearing mice, we found that stimulator cells from mice at TT were able to initiate T cell proliferation while those from mice at ST were not (Figure 5; tumor-bearing). These results suggest that the activated DCs found in 4T1 tumor-bearing mice at TT are more efficient antigen presenting cells than DCs from tumor-bearing ST mice as demonstrated by the superior ability of TT splenocytes to elicit T cell proliferation. As the *in vitro* portion of this work was all done at 37°C, these findings also suggest that cold stress can alter DC function over a prolonged period of time after DCs are removed from the mouse.

## Discussion

The relationships between stress and DC function are complex and depend upon the type and duration of stress, and whether the stressor is applied *in vivo* or *in vitro*. The type of DC (i.e., isolated from the bone marrow or skin) or stage of DC maturation when a stressor is encountered may also influence the impact of a particular stress (39). Additionally, DC function is dependent on the timing of antigen exposure and/or the type of antigen used, so these factors may also affect the observed relationship between stress and DC function (44).

In addition to summarizing some of the existing data on the effects of various stressors and stress hormones on DC function, we show here that the numbers and percentages of different subsets of DCs can be dependent upon housing temperature. Since sub-thermoneutral housing temperature is the standard condition under which mice are housed throughout the world, our data suggests that only using mice which are mildly cold stressed could be limiting our full understanding of the role of DCs in immune responses, including their role in anti-tumor immunity. Specifically, we have shown that tumor-bearing mice at ST have significantly more DCs compared to tumor-bearing mice at TT. However, the increased DCs seen at ST primarily display an immature phenotype (MHC II^−^CD86^−^) or they up-regulate MHC II but not CD86 rendering them unable to activate CD8^+^ T cells. The induction of signal 1 in the absence of signal 2 has been shown to lead to immune tolerance (12, 13). Thus, our studies suggest the potential for greater tolerance in mice at ST versus TT as splenocytes from ST mice were unable to activate T cell proliferation likely contributing to faster tumor growth. We observed enhanced T cell stimulatory ability by splenocytes from tumor-free mice at ST versus TT; however, when tumors were present the ability of ST splenocytes to activate T cells was diminished. While these data presented here is limited by the fact that we used whole splenocytes instead of isolated DCs to quantify the ability of cells from mice at ST and TT to activate T cells, the results presented strongly suggest that DCs from mice under mild cold stress are less able to undergo maturation prime T cells and elicit efficient T cell responses than mice maintained under thermoneutral conditions.

One possible explanation for the differences in tumor growth in mice from ST and TT is that DCs from mice at ST are more suppressive than those from mice at TT. It has been shown that a subset of murine DCs become particularly suppressive throughout tumor growth (100–103). Our previous observations suggest that when a tumor is present, the impact of cold stress is greatly exacerbated (55). This idea is further supported by these findings showing that T cell stimulation is greatly suppressed by splenocytes from mice at ST, but not TT.

The data presented here, along with other recent publications (81, 84–90) strongly suggest that the effects of chronic mild cold stress are important to consider when working with mouse models. Moreover, when studying the impact of experimentally induced stress, such as social or psychological stress, on DCs and other immune cells, it may be important to recognize that baseline data could be significantly influenced by inherent cold stress induced by standard housing conditions for laboratory mice.

## Future Research Questions

New questions emerge from the data presented here with regard to the effects of stress on DCs. Most importantly, what is the mechanism by which mild cold stress influences DC function? NE is involved in activation of thermogenesis in order to increase heat production to maintain normal body temperature (51, 85) and has already been strongly implicated for its roles in immunosuppression (20) and in regulating the polarization of macrophages (85). These observations strongly point to NE being a key player in the underlying relationship between cold stress and impaired DC function (55).

As mentioned earlier, cytokines affected by glucocorticoid treatment (43) or mild heating (60, 61, 63) include TNF-α, IFN-γ, IL-1α, IL-17, IL-10, and IL-12. How is the expression of these cytokines impacted by pre-existing mild cold stress in mice? In order to fully understand the impact of other types of stress in mouse models, it will be imperative to understand if cytokine production by DCs differs when mice are housed at sub-thermoneutrality compared to TT.

Here, we looked at DC expansion in response to inoculation with the 4T1 murine mammary carcinoma cell line. Do DCs in the presence of other tumor models respond similarly to cold stress? Similar analysis of mice at ST and TT using hematological tumors and other widely used cell lines representing different types of solid tumors, as well as human derived cell lines and patient xenograft models in immunosuppressed mice may elicit different findings regarding tumor growth control and DC function. Further, use of carcinogen-induced or transgenic mouse tumor models will all be important to establish the overall impact of cold stress on DC function.

We have shown that eliminating cold stress influences baseline properties of DCs in tumor-free and tumor-bearing mice. Therefore, a major question which should be addressed is how this may be influencing data interpretation of experiments in which additional stressors (such as social isolation) are imposed on pre-existing cold stress. It is also possible that previously demonstrated beneficial effects of mild hyperthermia on DC function could be related to the fact that control (non-heated) mice are actually cold stressed. In other words, applications of mild heat could have a similar effect on DCs as thermoneutural housing in which body temperature is not elevated. It is clear that the study of stress responses in mice should be done at more than one ambient temperature in order to understand the impact of this variable on data interpretation. Conducting experiments under thermoneutral conditions as well as sub-thermoneutral housing would help to eliminate the impact of pre-existent cold stress while studying the effects of other stressors on DC function.

In summary, since a complete understanding of DCs is critical for development of effective immunotherapies for cancer patients, it is essential to recognize that the function of these critical cells may be dependent upon ambient housing temperature and other factors which influence physiologic or metabolic stress experienced by laboratory mice used in preclinical studies.

## Materials and Methods

### Mice

Female, 8–10-week-old BALB/cAnNcr (BALB/c) and C57BL/6NCr (C57BL/6) mice were purchased from the NCI (Bethesda, MD, USA). Prior to experimentation, BALB/c mice were acclimated to ST or TT for 2 weeks.

### Mouse housing at ST and TT

Mice were maintained in specific pathogen-free facilities and were treated in accordance with the guidelines established by the IACUC at Roswell Park Cancer Institute (Buffalo, NY, USA). Cages containing Enrich-o’Cobs bedding (The Andersons, Inc., Maumee, OH, USA) housed mice 5 to a cage. Cages were held in Precision^®^ Refrigerated Plant-Growth Incubators (Thermo Scientific; Waltham, MA, USA) maintained at 22 or 30°C. Humidity was controlled using a Top Fin^®^ Air Pump AIR 1000 with Top Fin^®^ airline tubing.

### Cell line

4T1 murine mammary carcinoma cells were purchased from ATCC (Manassas, VA, USA). Cells were cultured in RPMI 1640 (Gibco, Grand Island, NY, USA) with 10% FBS, 10 mM l-glutamine, and 100 μg/ml penicillin/streptomycin. When cells reached ~90% confluence in culture, 1 × 10^4^ 4T1 cells were injected orthotopically into the fourth mammary fat pad of BALB/c, mice.

### Flow cytometry

Cells were collected from the spleen, tumor, and draining lymph node. Tissues were excised, washed, and filtered into a single cell suspension. Cells were counted with a hemocytometer and Trypan Blue solution. Cells were stained with Brilliant Violet 711™ anti-mouse CD4 (clone RM4-5; BioLegend; San Diego, CA, USA), Brilliant Violet 650™ anti-mouse CD3 (clone 17A2; BioLegend), Pacific Blue™ anti-mouse CDllb (clone M1/70; BioLegend), APC anti-mouse CD11c (clone N418; BioLegend), FITC anti-mouse CD86 (clone GL1; BD Pharmingen; San Jose, CA, USA), PerCp/Cy5.5 anti-mouse MHC (clone M5/114.15.2; BioLegend). Live cells were determined by staining cells with 4′,6-diamidino-2-phenylindole (DAPI; Life Technologies; Grand Island, NY, USA) and defined as DAPI-negative. Samples were analyzed on an LSRII flow cytometer (BD Pharmingen) and analyzed using FlowJo (Ashland, OR, USA) version 10.0.6.

### Mixed lymphocyte reactions

Spleens were excised from tumor-free and tumor-bearing BALB/c mice, and lymph nodes were excised from C57BL/6 mice. BALB/c splenocytes were irradiated at 30 Gy. BALB/c splenocytes (stimulator cells) and C57BL/6 lymphocytes (responder cells) were filtered, washed, and cultured at a ratio of one stimulator cell to two responder cells in 200 μl RPMI (10% FBS, 100 mM l-Glutamate, and 100 U/ml Penicillin–Streptomycin). After 72 h 1 μCi ^3^H-thymidine was added for 14–18 h. T cell proliferation was determined by ^3^H-thymidine incorporation.

### Data analysis and statistics

All data are presented as mean ± SEM All *p* values were determined using Student’s *t*-tests or two-way ANOVA with Bonferroni post-tests. All statistical analysis was completed using Prism software.

## Conflict of Interest Statement

The authors declare that the research was conducted in the absence of any commercial or financial relationships that could be construed as a potential conflict of interest.
